# Antivenom Production against *Bothrops jararaca* and *Bothrops erythromelas* Snake Venoms Using Cross-Linked Chitosan Nanoparticles as an Immunoadjuvant

**DOI:** 10.3390/toxins10040158

**Published:** 2018-04-16

**Authors:** Karla Samara Rocha Soares, Fiamma Gláucia-Silva, Alessandra Daniele-Silva, Manoela Torres-Rêgo, Nathália Kelly de Araújo, Yamara Arruda Silva de Menezes, Igor Zumba Damasceno, Denise Vilarinho Tambourgi, Arnóbio Antônio da Silva-Júnior, Matheus de Freitas Fernandes-Pedrosa

**Affiliations:** 1 Department of Pharmaceutical Sciences, Faculty of Pharmacy, Federal University of Rio Grande do Norte, Natal 59012-570, Brazil; karllasamara@yahoo.com.br (K.S.R.S.); fiammaglaucia@ufrn.edu.br (F.G.-S.); alessandradaniele@ufrn.edu.br (A.D.-S.); manoelatorres@ufrn.edu.br (M.T.-R.); nakar_rn@hotmail.com (N.K.d.A.); yamaramenezes@ufrn.edu.br (Y.A.S.d.M.); 2 Department of Materials Engineering, Technology Center, University Campus, Federal University of Rio Grande do Norte, Natal 59078-970, Brazil; igorzumba@ufrn.edu.br; 3 Laboratory of Immunochemistry, Instituto Butantan, São Paulo 05503-900, Brazil; denise.tambourgi@butantan.gov.br

**Keywords:** *Bothrops* venoms, antivenom, adjuvants, nanoparticles, chitosan, nanovaccines

## Abstract

In Brazil, envenomation by snakes of the genus *Bothrops* is clinically relevant, particularly for the species *Bothrops jararaca* and *B. erythromelas*. The most effective treatment for envenomation by snakes is the administration of antivenoms associated with adjuvants. Novel adjuvants are required to reduce side effects and maximize the efficiency of conventional serum and vaccine formulations. The polymer chitosan has been shown to have immunoadjuvant properties, and it has been used as a platform for delivery systems. In this context, we evaluated the potential immunoadjuvant properties of chitosan nanoparticles (CNPs) loaded with *B*. *jararaca* and *B. erythromelas* venoms in the production of sera against these venoms. Stable CNPs were obtained by ionic gelation, and mice were immunized subcutaneously for 6 weeks with 100 µL of each snake venom at concentrations of 5.0 or 10.0% (*w*/*w*), encapsulated in CNPs or associated with aluminium hydroxide (AH). The evaluation of protein interactions with the CNPs revealed their ability to induce antibody levels equivalent to those of AH, even with smaller doses of antigen. In addition, the CNPs were less inflammatory due to their modified release of proteins. CNPs provide a promising approach for peptide/protein delivery from snake venom and will be useful for new vaccines.

## 1. Introduction

Snakebite envenoming is a highly relevant global public health issue that is considered a major occupational health problem and has been a perennial cause of death or chronic disability for many active young people [1,2]. The global incidence of snakebites is approximately 5,400,000 per year, and approximately 125,000 of these cases are fatal [1,3]. Snakebite envenoming is estimated to occur mostly in Asia, Africa, and Latin America, where between 20,000 and 94,000 fatalities are reported each year [2,3,4]. Generally, snakebites are most common in tropical and subtropical areas, but they also occur in regions with temperate climes [3]. In Brazil, accidental snakebite envenoming represents an important public health hazard. Ninety percent of the yearly estimated 20–30,000 snakebite accidents are caused by species of the *Bothrops* genus. *B. jararaca* (South and Southeast), *B. moojeni* (Centre-West), *B. atrox* (North), and *B. erythromelas* (Northeast) are responsible for most of these accidents [5,6,7,8,9,10].

Systemic signs of envenomation are observed in patients bitten by *Bothrops* snakes, such as haemostatic disturbances, signs at the site of bite, haemorrhage, myonecrosis, dermonecrosis, and inflammatory reactions (oedema, leukocyte infiltration, and pain) [9,11,12,13]. The pathogenesis of *Bothrops* envenomation is complex, but local manifestations have been directly associated with the activity of metalloproteases, phospholipase A_2_, or serine proteases present in the venom [11,12,14,15,16]. Parenteral administration of animal-derived antivenoms is the mainstay treatment for snakebite envenoming [17,18,19,20]. In Brazil, the treatment for envenomation by bothropic species involves the use of an equine polyspecific antibothropic (or antibothropic-lachetic) F(ab’)2 antivenom, prepared by conventional immunization schedules using a pool that includes the venoms of five *Bothrops* species: *B. jararaca, B. jararacussu, B. moojeni, B. alternatus*, and *B. neuwiedi* [10,17].

The serums routinely produced are safe and effective for treatment. However, some problems have arisen related to the application of immunoadjuvants for vaccines and sera, such as the toxic potential and collateral effects of these formulations [21,22,23]. Aluminium-containing adjuvants are approved by the United States Food and Drug Administration for human use and are widely applied in immunobiological production [23]. There are two main aluminium containing adjuvants: aluminium hydroxide (AH) and aluminium phosphate [23].

Unfortunately, some evidence about the toxicity of aluminium salts has been reported [24,25,26,27]. The effects of these salts include late hypersensitivity [28,29], severe granulomatous inflammation [22,27,30] and pruritic subcutaneous nodules [28]. Other side effects of aluminium include increased immunoglobulin E titres, allergenicity, and potential neurotoxicity [22,30,31]. Normally, aluminium is excreted by the kidneys; however, under certain conditions, such as reduced renal function, aluminium accumulates in the body and can become toxic [30].

Consequently, several studies have focused on the development of new adjuvants for human vaccines to generate stronger vaccines capable of inducing protective and long-lasting immunity in humans with reduced side effects and toxicity compared with conventional formulations [30]. For example, one study evaluated the development of a new carrier for vaccine delivery that was aimed at treating scorpion envenoming [32]. Sustained drug release through nanocarriers has already been introduced in previous studies [33]. Hydrophilic nanoparticles have received much attention regarding their ability to deliver therapeutic peptides, proteins, antigens, oligonucleotides, and genes by intravenous, oral, and mucosal administration routes [34,35]. For protein incorporation, the ideal diameter of a nanoparticle should be between 100 and 200 nm [21,36,37]. Nanoparticles can potentially circulate for a long time in the body, releasing their loads over multiple hours or days, depending on their properties [33,38,39]. Hence, protein delivery through nanoparticles is an effective way to control drug release as well as to design an efficient protein delivery system [33]. In this context, several polymers that have shown interesting properties, such as chitosan, have been investigated as both delivery systems and adjuvants for vaccine preparations [34,40,41,42].

Chitosan (CN) is a naturally occurring polysaccharide obtained by partial N-deacetylation of chitin. It is used as a nanocarrier because of its unique biodegradability, biocompatibility, hydrophilicity, non-toxic, and immunomodulatory properties as well as its low cost [34,35,41,43,44,45]. CN has been extensively investigated for carrier and delivery system formulations for therapeutic macrosolutes. Particularly, gene and protein molecule formulations have been explored due to the positive charge of chitosan, which can be easily complexed with negatively charged DNAs and proteins [21,46,47]. Chitosan can effectively bind DNA and proteins, protecting these molecules from nuclease and protease degradation, respectively [47].

Effective immune responses have been observed when chitosan was used as an immunoadjuvant in vaccines for immunization against *Helicobacter pylori* [48], diphtheria [49], hepatitis B [50], *T. serrulatus* venom [21], and *Naja naja oxiana* snake venom [36]; moreover, CN has recently been applied in gene therapy strategies for the control of *Aedes aegypti* mosquito proliferation [43].

A more detailed approach to the use of this non-toxic and non-inflammatory immunoadjuvant as a delivery system would provide a powerful public health tool as well as an innovative approach for the development of a new serum against toxins from the *B. jararaca* and *B. erythromelas* snake venoms. This system could generate an effective immune response with low loads of antigens, thus minimizing the adverse effects caused by traditional immunoadjuvants. This study evaluated the potential immunoadjuvant effects of chitosan nanoparticles (CNPs) loaded with *B*. *jararaca* and *B. erythromelas* venoms in the production of serum against these venoms, and comparatively assessed the inflammatory process generated by CNPs *versus* a conventional adjuvant.

## 2. Results

### 2.1. Preparation of Chitosan Nanoparticles

Titrations of a tripolyphosphate (TPP) solution into a chitosan solution formed cross-linked chitosan nanoparticles spontaneously through intra- and intermolecular bonds between the phosphate groups of the polyanion TPP and the amine groups of chitosan [35,51,52]. The chitosan nanoparticles were generated via an ionic gelation technique and had an average size of 167.5 nm, a zeta potential (ZP) of +24.5 mV, and a polydispersity index (PdI) of ≤0.3 (Table 1). The ZP and PdI values of different samples were not significantly different.

### 2.2. Protein Loading Efficiency of the Bothrops jararaca and Bothrops erythromelas Venoms

The snake venom proteins were loaded into the nanoparticles using an incorporation method in which the proteins were dissolved in the TPP solution. The proteins did not impair the formation of the chitosan nanoparticles, which occurred spontaneously during the dropwise addition of TPP into the chitosan solution.

The successful production of cross-linked nanoparticles was confirmed by their average particle size. A slight increase in particle size occurred with the addition of venoms into the system, but a small size of 200 nm was maintained. The CNPs loaded with both *B. jararaca* and *B. erythromelas* venom exhibited similar behaviour. All formulations had a polydispersity index of ≤0.3 (with no significant differences among the samples), and the protein loading did not change their cationic characteristics (Table 1).

The nanoparticles demonstrated a great protein loading capacity with the ability to maintain a particle size of approximately 200 nm for all of the different venom and chitosan ratios used. The data showed an encapsulation efficiency greater than 87% for all tested formulations containing *B. erythromelas* venom. For *B. jararaca*, the protein encapsulation efficiency reached levels greater than 67% (Table 1). Electrophoresis assays of protein-free nanoparticles and protein-loaded nanoparticles were used to visualize these protein loading events.

**Figure 1:**
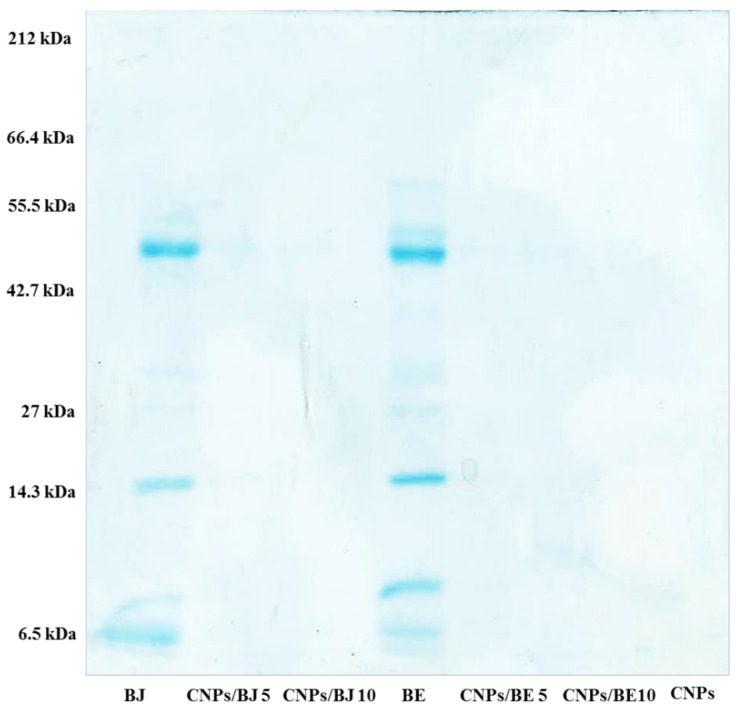
Electrophoretic profile of *Bothrops jararaca* venom at 10% (BJ), chitosan nanoparticles with *Bothrops jararaca* at 5% (CNPs/BJ 5) and 10% (CNPs/BJ 10), *Bothrops erythromelas* venom at 10% (BE), chitosan nanoparticles with *Bothrops erythromelas* venom at 5% (CNPs/BE 5) and 10% (CNPs/BE 10), and CNPs.

The protein profiles of the *B. jararaca* and *B. erythromelas* venoms as well as the distinct protein-loaded cross-linked chitosan nanoparticles were analysed by SDS-PAGE and are shown in Figure 1. The *B. jararaca* (BJ) and *B. erythromelas* (BE) venom protein fractions were distributed within a molecular mass range of approximately 14 to 66.4 kDa. A pool of proteins was characterized for medium to low molecular weight proteins. Highlighting the bands between 55.5 and 42.7 kDa, as well as those at 14.3 and 6.5 kDa, was possible in both venoms analysed. Comparing the electrophoretic profiles of the protein-free nanoparticles, the free venom proteins (BJ and BE), and the protein-loaded nanoparticles with the *B. jararaca* protein-loaded nanoparticles (CNPs/BJ 5 and CNPs/BJ 10) and the *B. erythromelas* protein-loaded nanoparticles (CNPs/BE 5 and CNPs/BE 10) demonstrated the ability of the CNPs to be a nanocarrier for the examined negatively charged proteins. The bands of venom proteins were not detected in the nanoparticle samples, thus confirming formation of venom protein–nanoparticle complexes (Figure 1, lanes CNPs/BJ 5, CNPs/BJ 10, CNPs/BE 5, CNPs/BE 10 and CNPs). These results reinforce the high protein–nanoparticle loading capacity of the CNPs.

**Figure 2:**
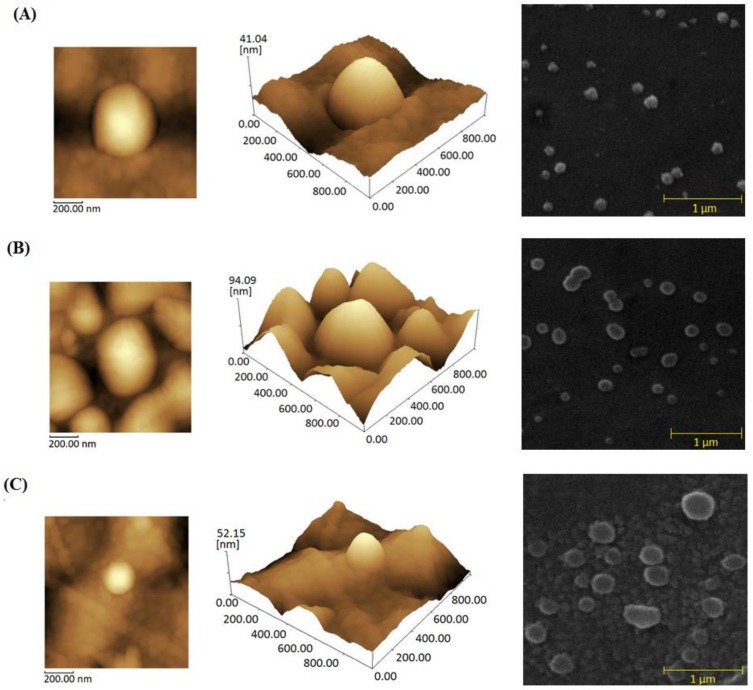
2D and 3D atomic force microscopy and scanning electron microscopy images of *Bothrops* protein-loaded chitosan nanoparticle formulations. (**A**) Protein-free chitosan nanoparticles, (**B**) *Bothrops jararaca* protein-loaded chitosan nanoparticles at a concentration of 10% and (**C**) *Bothrops erythromelas* protein-loaded chitosan nanoparticles at a concentration of 10%.

The morphology of the nanoparticles and protein-loaded nanoparticles was examined using atomic force microscopy (AFM) and scanning electron microscopy (SEM). Their surfaces and aspects are shown in Figure 2; they presented a size of approximately 200 nm with a spherical shape, homogeneous aspect, rough surface, and great encapsulation efficiency.

**Figure 3:**
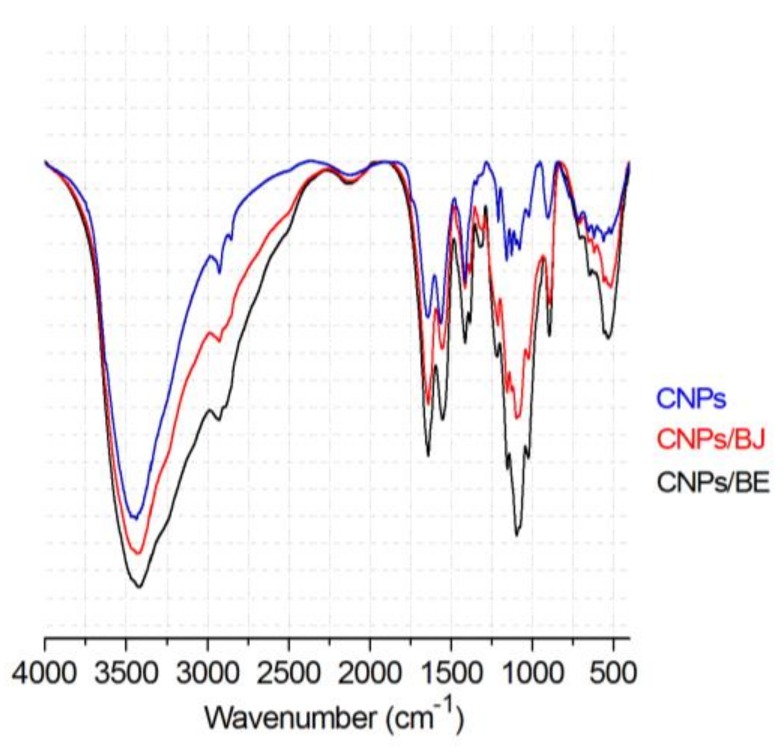
Infrared spectroscopy of CNPs, chitosan nanoparticles loaded with *Bothrops jararaca* venom protein (CNPs/BJ) and chitosan nanoparticles loaded with *Bothrops erythromelas* venom protein (CNPs/BE).

Fourier transform infrared (FT-IR) spectroscopy analyses were used to evaluate the interactions between chitosan and the venom proteins. This evaluation was based on the fact that this interaction leads to frequency shifts or absorption band splitting [21]. Figure 3 shows the spectra recorded for the CNPs and protein–protein nanoparticles containing distinct snake venoms loaded at a 10% *w*/*w* concentration. In the analysis of the CNPs, N-H bending and C=O stretching of amide groups were observed at 1561 and 1649 cm^−1^, respectively, indicating pure chitosan [53]. Moreover, the higher intensity of these bands in the CNPs/BJ and CNPs/BE is due to the formation of new amide interactions by ionic gelation. This fact is further supported by the remarkable P=O band stretching at 1214 cm^−1^ and the P-O-P stretching at 892 cm^−1^, which is characteristic of TPP [53,54]. These characteristic intermolecular interactions have been established in previous studies of ionic gelation and are due to chitosan chain cross-linking [55,56]. The protein-loaded nanoparticle sample had C=O bands of increased intensity for the carbonyl groups of the secondary amides (1651 cm^−1^) and primary amides (1540 cm^−1^) of proteins.

### 2.3. Stability Assay

**Figure 4:**
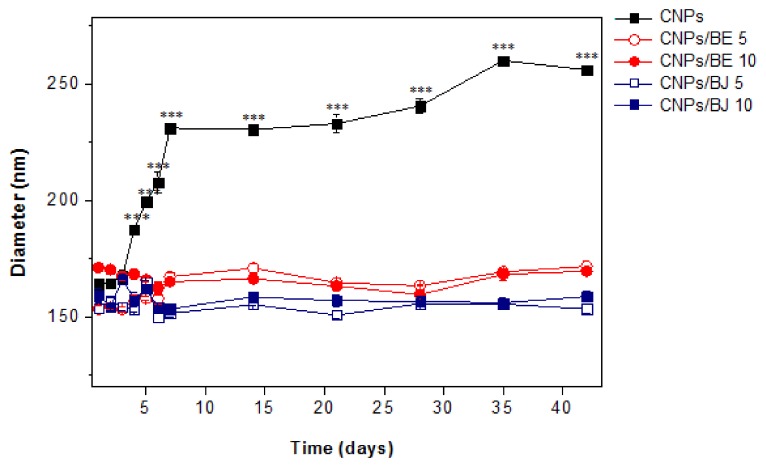
Evaluation of the stability of cross-linked chitosan nanoparticle samples loaded with *Bothrops jararaca* or *Bothrops erythromelas* venom (5% and 10% *w*/*w*). *** *p* < 0.001 compared to the size of the CNPs on day 1. CNPs: protein-free chitosan nanoparticles; CNPs/BE 5: chitosan nanoparticles loaded with *B. erythromelas* protein at a concentration of 5%; CNPs/BE 10: chitosan nanoparticles loaded with *B. erythromelas* protein at a concentration of 10%; CNPs/BJ 5: chitosan nanoparticles loaded with *B. jararaca* protein at a concentration of 5%; CNPs/BJ 10: chitosan nanoparticles loaded with *B. jararaca* protein at a concentration of 10%.

The physical stability of the protein-loaded nanoparticles and protein-free nanoparticles was assessed using samples stored at 4 ± 2 °C for 40 days (Figure 4). Particle size measurements, which were taken every 5 days, revealed the incredible performance of the protein-loaded CNPs. Venom loading improved the stability of the CNPs, as nanoparticles without added venom began to increase in size after the fifth day (*** *p* < 0.001). No differences were observed among the protein-loaded nanoparticle formulations.

### 2.4. Antibody Titres

Mice were immunized subcutaneously for 6 weeks with 100 µL of each snake venom at concentrations of 5.0 or 10.0% (*w*/*w*), encapsulated in CNPs or associated with AH [21]. One week after booster vaccination, serum was obtained via ocular plexus blood collection. The serum samples were subjected to serial dilutions with a standard diluent (PBS/0.1% *w*/*w* BSA), starting at a dilution of 1:6400 and continuing to 1:204,800, and the antibody titres were evaluated by enzyme-linked immunosorbent assay.

**Figure 5:**
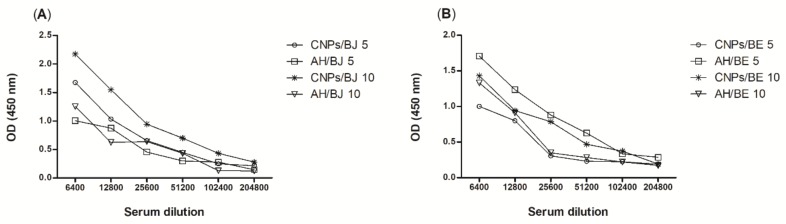
Evaluation of antibody titres from mice immunized subcutaneously for 6 weeks with *Bothrops jararaca* (**A**) or *Bothrops erythromelas* (**B**) venom at concentrations of 5.0 or 10.0% encapsulated in CNPs or associated with AH, as determined by enzyme-linked immunosorbent assays.

The antibody titres were detected in the serum of mice immunized with venom from both snakes, *B. jararaca* (Figure 5A) and *B. erythromelas* (Figure 5B), up to the 1:102,400 dilution. These results were statistically equal to those of the AH immunized groups and demonstrated that the nanoparticles can stimulate refined and high titres of production even at low concentrations. Similar results were found in experiments performed with cross-linked chitosan nanoparticles loaded with protein from the scorpion *Tityus serrulatus* [21].

## 3. Discussion

In the present study, cross-linked chitosan nanoparticles were successfully produced using the established parameters of the ionic gelation technique. Chitosan nanoparticles containing *B. jararaca* or *B. erythromelas* venom formed spontaneously when a solution of protein and TPP was added to a chitosan solution. In the incorporation process, the proteins are entrapped and embedded into the chitosan–protein matrix, with some protein molecules also adsorbing to the particle surface [51,57]. This phenomenon explains the high encapsulation efficiency of nanoparticles of <200 nm, even after the addition of snake venom. This fact is further supported by the intermolecular interactions of the carboxyl groups from proteins with the free amine groups of chitosan observed in the FT-IR spectra. These specific interactions can be monitored by the enhancement of the intensities of these bands in the spectra of protein-loaded nanoparticles. The ionic gelation technique has been successfully applied to obtained chitosan nanoparticles for medical proposes, such as anti-staphylococci agents [58] and letrozole delivery (anticancer drug) [59].

Loading protein into nanoparticles preserves their physical stability by keeping their average diameter in the nanometre range and their particle size distribution uniform for parenteral administration [60]. Their characteristics, including an average size of 167.5 nm, a zeta potential of +24.5 mV, and a polydispersity index of ≤0.3, are in accordance with other chitosan nanoparticles obtained in other studies. Costa et al. (2017) reported an average size of 244 (±11.64) nm with a polydispersity index of 0.358 and a zeta potential of +17.3 9 (±1.41) mV [58]. The FT-IR spectra recorded for the chitosan nanoparticles are consistent with previously published data by our group [54]. These results confirm the presence of pure chitosan as well as new amide interactions from the ionic gelation process. Costa et al. (2017) related that chitosan–TPP crosslinking is realized through interactions between TPP and the amino groups present in chitosan, which can be confirmed by the peak at 1586 cm^−1^ [58]. Alterations in the NH_2_ bending vibration band of chitosan were also described by Gomathi and colleagues [59]. In addition, the experimental data demonstrated that the electrostatic interactions of the venom proteins with chitosan also induce crosslinking, which is a barrier for rapid protein release in the medium.

The high encapsulation efficiency (greater than 87% for all tested *B. erythromelas* protein concentrations) observed for the cross-linked chitosan nanoparticles can be attributed to venom dissolution in the TTP solution. Thus, these venom proteins are fully trapped inside the polymer nanomatrix at the instant of nanoparticle cross-linking. Furthermore, that *Bothrops* venom has negatively charged peptides [9,61] can also explain the high loading efficiency. These results demonstrate the substantial ability of the optimized nanoparticle formulation to carry negatively charged proteins, maintaining colloidal stability, which is not common for particle-encapsulated proteins. The 3D conformational structure of nanoparticles is complex and dependent on environmental conditions, such as pH, ionic strength, electrostatic interactions and composition. The great ability of this polymeric nanocarrier to interact with negatively charged biomolecules is due to the cationic character of chitosan [54,60,62].

An effective polymeric nanocarrier should be able to condense DNA or protein to protect against denaturation [63]. This ability of cross-linked chitosan nanoparticles was confirmed for distinct proteins via the gel retardation assay. The data revealed that when nanoparticle–protein complexes formed, the protein bands were no longer visible, indicating entrapment of the venom protein in the polymeric matrix.

The increased physical stability of the CNPs induced by protein loading suggests a possible perturbation of the electrostatic interactions among the positively charged groups of chitosan during the formation of nanoparticles that resulted in further steric stabilization [47,51].

A suitable antigen-presenting system is required for vaccine success and is directly related to the adjuvant or vehicle used [48]. The treatment for envenomation by bothropic species includes antiophidic serum administration. Although this approach has been efficient, studies must be performed to identify a less toxic adjuvant that is able to induce antibody titres greater than those produced by the already established approach.

Mice were immunized with the chitosan nanoparticle or AH adjuvant alone or in conjunction with *B. jararaca* or *B. erythromelas* venom. In terms of immune protection, the data revealed that animals immunized with CNPs cross-linked with *B. erythromelas* venom at concentrations of 5.0 or 10.0% were statistically equal when compared with the antibody titres of groups vaccinated with the adjuvant AH and the venom at the same concentrations. For animals immunized with CNPs loaded with *B. erythromelas* venom, the antibody titres were not significantly different when compared with those of the AH groups immunized with the same venom. The application of CNPs as an immunoadjuvant in vaccines can support an efficient immune response and may lead to antibody production similar to that induced by aluminium hydroxide. However, this polymer displays the advantage of being less inflammatory or non-inflammatory and provides modified antigen release, which promotes greater antibody titres in serum with the administration of a smaller amount of antigen.

The experimental data demonstrated that chitosan nanoparticles can induce antibody production equivalent to that of AH, reinforcing data previously published by our group [21]. Other studies have shown efficient immunization using CNPs; for example, the strong adjuvant effect of γ-PGA and chitosan nanoparticles for toxin B from *Clostridium difficile* was reported [64]. Thus, chitosan nanoparticles provide an efficient and secure approach for the delivery of peptides/proteins from snake venom.

## 4. Materials and Methods

### 4.1. Materials

Chitosan (85% deacetylated; molecular weight, 90–190 kDa), AH, and TTP were purchased from Sigma-Aldrich^®^ (Saint Louis, MO, USA). The *B. jararaca* and *B. erythromelas* venoms were kindly supplied by the Butantan Institute in São Paulo. The bicinchoninic acid (BCA) Protein Assay kits were purchased from Pierce (Woburn, MA, USA), and the Mouse IgG total ELISA kits were purchased from eBioscience (San Diego, CA, USA). All other reagents and solvents used were of analytical grade.

### 4.2. Cross-Linked Chitosan Nanoparticles

The cross-linked chitosan nanoparticles used for the incorporation of the *B. jararaca* and *B. erythromelas* venoms were acquired using the ionic gelation technique. A solution of 0.1% *w*/*v* TPP in water obtained by reverse osmosis (<1.3 µS cm^−1^) was added dropwise to chitosan (0.1% *w*/*v* in 0.175% *w*/*v* acetic acid) under magnetic stirring. After an opalescent suspension spontaneously formed, the mix was maintained under agitation at room temperature for 30 min.

### 4.3. Loading of the B. jararaca and B. erythromelas Venoms into Cross-Linked Chitosan Nanoparticles

To load protein into the chitosan nanoparticles, different amounts of proteins (*B. jararaca* or *B. erythromelas* venoms) in different proportions (5, 10 and 15% *w*/*w*) were solubilized in the TPP solution in a pre-mixing procedure under magnetic stirring (700 rpm) in a thermostatized bath at 20 ± 2 °C [51]. These solutions containing TPP and each specific protein sample were titrated into the copolymer solution using the same optimized procedure described above to obtain cross-linked chitosan nanoparticles. Protein-free nanoparticles were also produced to evaluate the effect of protein loading on the physio-chemical properties of the particles. All experimental procedures were carried out in triplicate, and the data is expressed as the mean ± standard deviation (SD).

### 4.4. Protein-Loading Efficiency Assay

Distinct samples of protein-loaded (with the *B. jararaca* and *B. erythromelas* venoms) cross-linked chitosan nanoparticles were carefully transferred to 1.5-mL centrifuge tubes and then centrifuged at 20,000× *g* for 30 min at 4 °C. The protein concentration of each supernatant was analysed using a BCA Protein Assay Kit according to the manufacturer’s instructions. The encapsulation efficiency (EE) was calculated using Equation (1) [47,60]. All analyses were carried out in triplicate, and the data isexpressed as the mean ± standard deviation (SD).

(1) $$\mathrm{EE}\%=\frac{\left(Total\;protein-\mathrm{supernatant}\;\mathrm{protein}\right)}{Total\;protein}\times 100$$

### 4.5. Electrophoresis

Sodium dodecyl sulfate-polyacrylamide gel electrophoresis (SDS-PAGE) was used to determine the protein profiles of the *B. jararaca* and *B. erythromelas* venoms and their resultant protein-loaded cross-linked chitosan nanoparticles. For this, a mini-gel electrophoresis system (Mini-Protean^®^ II, BIO-RAD, Hercules, CA, USA) was used [65]. The migration of a standard protein mixture (Gibco-BRL Life Technologies, Gaithersburg, MD, USA) was used to determine the relative molecular masses of the test proteins. The obtained gels were stained with a solution of Coomassie Brilliant Blue R-250 [66].

### 4.6. Physicochemical Properties of the Nanoparticles

Scanning electronic microscopy images (SSX550, Shimadzu, Tokyo, Japan) and atomic force microscopy (AFM, SPM-9700, Shimadzu, Tokyo, Japan) were used to assess the shape and surface aspects of the nanoparticles. For the AFM analysis, one drop of each nanoparticle dispersion was placed on a washed microscope slide and dried under a desiccator for 24 h prior to observation. The measurements were performed at room temperature in cantilever non-contact mode.

The physicochemical properties of the CNPs such as their zeta potential (ZP) and polydispersity index (PdI) were determined. The potential of the slipping/shear plane of a colloid particle moving under an electric field corresponds to the ZP. The electric double layer (EDL) of electrophoretically mobile particles and the layer of dispersant around them show different potential, which is the ZP [67]. The PdI provides information about the homogeneity of the particle size distribution in a sample [68].

Particle size measurements of distinct nanoparticles were assessed at 25 °C using a cumulative method of analysis in which the intensity of the light scattered (DLS) was measured in a particle size analyser (Zeta Plus-Brookhaven Instruments, New York, NY, USA) at a wavelength of 659 nm with a 90° detection angle. The correlation was run in parallel mode, and data was analysed using Zeta Plus^®^ Particle Sizing version 3.95 software. Zeta potential (ZP) measurements were performed using the same equipment by applying a field strength approximately 5.9 V cm^−1^. Five runs were performed for each sample to determine the ZP value with PALS Zeta Potential Analyser software using electrophoretic mobility according to the Helmholtz-Smoluchowski equation. The samples were diluted 1:100 (*v*/*v*) with purified water. All measurements were carried out in triplicate, and the data is expressed as the mean ± standard deviation (SD) [21,54].

Fourier transformed infrared absorption spectra (FT-IR) were recorded using a Prestige 21 FT-IR spectrophotometer (Shimadzu, Tokyo, Japan). Spectra of cross-linked chitosan nanoparticles were compared with protein-loaded cross-linked chitosan nanoparticles containing the *B. jararaca* and *B. erythromelas* venoms. The samples were dried using a Centrivap Labconco speed vacuum concentrator (Kansas City, MO, USA), mixed with potassium bromide (KBr) in a melting pot and then compressed in hydraulic press. The weight ratio between the powder and KBr was approximately 1:200 *w*/*w* [21,54].

### 4.7. Stability Assay

Samples of protein-loaded nanoparticles containing the *B. jararaca* and *B. erythromelas* venoms (5 and 10% *w*/*w*) were stored at 20 ± 2 °C for 40 days; every 5 days, the particle diameters were assessed at 25 °C using a cumulative method of analysis in which the intensity of the light scattered (DLS) was measured in a particle size analyser (Zeta Plus-Brookhaven Instruments, New York, NY, USA) at a wavelength of 659 nm with a 90° detection angle. The correlation was run in parallel mode, and the data was analysed using Zeta Plus^®^ Particle Sizing version 3.95 software.

### 4.8. Animals

Male and female BALB/c mice (25–35 g, 6–8 weeks of age) were received from the “Animal Facility at the Center for Health Sciences” at the Federal University of Rio Grande do Norte (UFRN). The animals were kept under controlled temperature (22 ± 2 °C) conditions with free access to commercial feed and water *ad libitum*, and they spent at least one week in the experimental room prior to the adaptation test. The animal care and experimental assays were performed in accordance with the guidelines established for the care of laboratory animals (Committee for Ethics in Animal Experimentation at the Federal University of Rio Grande do Norte, Protocol No: 003/2012; 18 April 2012).

### 4.9. Immunization

One hundred microliters of the *B. jararaca* and *B. erythromelas* venoms at different concentrations (5 or 10% *w*/*w*) cross-linked with the CNPs or combined with AH were used to immunize the animals. Immunization was performed six times, once *per* week, via the subcutaneous administration of the test compounds in the lumbar region (bilaterally) [21]. As a control, animals were immunized with the adjuvants in the absence of either venom.

### 4.10. Serum Production

Blood samples were treated for clot retraction. Initially, samples were kept at 37 °C for 30 min. After this step, the samples were incubated at 4 °C for 2 h and then centrifuged at 15,000× *g* for 5 min at 4 °C. The serum (supernatant) was collected and maintained at −20 °C [21].

### 4.11. Serum Antibody Responses

Serum samples were subjected to serial dilutions with a standard diluent (phosphate buffer saline (PBS)/ 0.1% *w*/*w* bovine serum albumin (BSA)), starting at a 1:25 dilution and continuing to 1:204,800, and antigen-specific serum antibody reactions were determined by enzyme-linked immunosorbent assays (ELISAs).

The ELISA assays were performed using the protocol described by Fernandes-Pedrosa in 2002 [69]. Each plate was sensitized with 100 μL/well of a venom solution (10 µg/mL *w*/*v*), followed by incubation overnight at 4 °C. After, the wells were washed (PBS solution), blocked with 100 μL of blocking solution (PBS/5% *w*/*w* BSA), and incubated at 37 °C for 2 h. The blocking solution was discarded, and 100 μL/well of each pre-diluted serum sample in PBS/0.1% *w*/*w* BSA were added and then incubated at 37 °C for 1 h. After washing three times, conjugated-antibodies were added (100 μL/well), and the plate was incubated at 37 °C for 1 h. Subsequently, the plate was washed, and 50 μL/well of diluted detection antibodies were added, followed by incubation at room temperature for 3 h. The plate was then washed again, the substrate was added, and the plate was incubated at room temperature for 15 min. Finally, the plate was read at 450 nm in a microplate reader.

### 4.12. Statistical Analysis

The results are expressed as the mean ± standard deviation. Statistical analyses were carried out using Student’s t tests or one-way analysis of variance (ANOVA) with Tukey’s tests using GraphPad Prism version 5.00 (San Diego, CA, USA). Differences in the mean values with *** *p* < 0.001, ** *p* < 0.01 or * *p* < 0.05 were considered statistically significant.

## 5. Conclusions

Data presented here demonstrates that cross-linked CNPs obtained via an ionic gelation technique presented high efficiency encapsulation for all formulations, which was confirmed by electrophoretic profiling and encapsulation efficiency assays. The nanoparticles were highly stabile for 40 days, and the addition of venom to the nanoparticles improved their stability. Considering the antibody levels produced, the results demonstrate that CNPs perform equivalent to or better than AH as an immunoadjuvant, yet chitosan is a less inflammatory biopolymer that requires a smaller dose of antigen, likely due to its modified release. This study presents a new immunization adjuvant approach for snake venom that could be applied to obtain polyclonal serum. As a follow-up to the development of this approach, our research group intends to use the CNPs in large mammals to evaluate the scaled-up production of antivenom serum. This system could be used in the future to obtain pharmaceutical composites that are very useful for public health.

## Figures and Tables

**Table 1:** Physicochemical properties of different cross-linked nanoparticles containing *B. jararaca* (BJ) and *B. erythromelas* (BE) venoms at concentrations of 5, 10, and 15%.

Sample	Size (nm)	Zeta Potential (mV)	PdI	Encapsulation Efficiency (%)
CNPs	159.6 ± 2.2	24.50 ± 3.64	0.272 ± 0.01	-
CNPs/BJ 5	179.3 ± 9.4 **	23.03 ± 3.09	0.188 ± 0.08	76.7
CNPs/BJ 10	174.7 ± 5.0 **	24.91 ± 2.91	0.203 ± 0.07	67.7
CNPs/BJ 15	187.3 ± 7.5 ***	31.40 ± 3.86	0.185 ± 0.07	74.0
CNPs/BE 5	189.4 ± 1.0 ***	20.71 ± 2.93	0.305 ± 0.05	97.2
CNPs/BE 10	160.0 ± 2.3	19.00 ± 2.76	0.302 ± 0.01	87.6
CNPs/BE 15	200.3 ± 5.6 ***	27.21 ± 1.50	0.328 ± 0.05	88.1

Values are the mean ± standard deviation (S.D.), *n* = 3; *** *p* < 0.001 and ** *p* < 0.01 for the venom-group compared to the CNP group.
